# The relationship between liver histology and thyroid function tests in patients with non-alcoholic fatty liver disease (NAFLD)

**DOI:** 10.1371/journal.pone.0249614

**Published:** 2021-04-06

**Authors:** Roberta D'Ambrosio, Irene Campi, Marco Maggioni, Riccardo Perbellini, Enza Giammona, Roberta Stucchi, Marta Borghi, Elisabetta Degasperi, Annalisa De Silvestri, Luca Persani, Laura Fugazzola, Pietro Lampertico

**Affiliations:** 1 Division of Gastroenterology and Hepatology, Foundation IRCCS Ca’ Granda Ospedale Maggiore Policlinico, Milan, Italy; 2 Department of Endocrine and Metabolic Diseases, IRCCS Istituto Auxologico Italiano, Milan, Italy; 3 Foundation IRCCS Ca’ Granda Ospedale Maggiore Policlinico, Division of Pathology, Milan, Italy; 4 Division of Laboratory Medicine, IRCCS Istituto Auxologico Italiano, Milan, Italy; 5 Clinical Epidemiology and Biometric Unit, Foundation IRCCS Policlinico San Matteo, Pavia, Italy; 6 Department of Clinical Sciences and Community Health, University of Milan, Milan, Italy; 7 Department of Pathophysiology and Transplantation, CRC “A.M. e A. Migliavacca” Center for Liver Disease, University of Milan, Milan, Italy; Medizinische Fakultat der RWTH Aachen, GERMANY

## Abstract

**Background:**

Data on the role of hypothyroidism in the pathogenesis of non-alcoholic fatty liver disease (NAFLD) and liver fibrosis are conflicting, although selective Thyroid Hormone Receptor (THR)-β agonists have been identified as potential therapy in patients with non-alcoholic steatohepatitis (NASH). Therefore, we investigated the association between hypothyroidism and NAFLD histological features potentially associated with progressive liver disease.

**Methods:**

Between 2014 and 2016, consecutive patients with histologically proven NAFLD and frozen serum available for thyroid function tests assessment were included. NAFLD was staged according to the NAFLD Activity Score (NAS), and fibrosis according to Kleiner. NASH was defined as NAS ≥4, significant fibrosis as F2-F4 and significant steatosis as S2-S3. Thyroid function tests (TFT; TSH, FT3, FT4, rT3), TPO-Ab and Tg-Ab were also assessed.

**Results:**

Fifty-two patients were analyzed: median age 54 years, 58% females, LSM 7.8 kPa, 27% diabetics, 14% hypothyroid. At histology, NASH was present in 21 (40%), F2-F4 in 28 (54%) and S2-S3 in 30 (58%) patients. Rates of hypothyroidism were similar independently of the presence of NASH (p = 0.11), significant fibrosis (p = 0.21) or steatosis (p = 0.75). However, hypothyroid patients displayed a higher NAS (p = 0.02) and NASH (p = 0.06) prevalence. At multivariate analysis, TFT were not independently associated with histology.

**Conclusion:**

Hypothyroidism was highly prevalent in NAFLD patients, and was associated with increased NAFLD activity, but not with fibrosis and steatosis severity. Thus, thyroid dysfunction might play a direct and/or indirect in the pathogenesis of NAFLD and NASH.

## Introduction

Non alcoholic fatty liver disease (NAFLD) is an emerging chronic liver disease, with an estimated prevalence of 17–46% in Western Countries **[1]**. NAFLD is defined by the presence of steatosis within the liver, and its histological spectrum ranges from steatosis alone (NAFL; *non alcoholic fatty liver*) to non-alcoholic steatohepatitis (NASH). This latter condition may ultimately lead to cirrhosis, the main risk factor for liver-related complications, such as hepatocellular carcinoma (HCC), portal hypertension and end-stage liver disease (ESLD) **[2,3]**.

Several conditions have been associated with NAFLD, such as diabetes, which is the strongest predictor of NASH and liver fibrosis **[4]**, insulin resistance (IR), dyslipidemia and obesity. More recently, hypothyroidism has been identified as a probable risk factor for NAFLD **[5–7]**. Thyroid Hormone (TH) influences both lipid and carbohydrate metabolism **[8,9]**. Indeed, most of key genes involved in these metabolic pathways are regulated by TH *via* the Thyroid Hormone Receptor-β (THR-β) which is the main isoform expressed in the liver **[10,11].** Hypothyroidism reduces lipolysis and decreases gluconeogenesis with consequent impairment of triglyceride clearance and, β-oxidation of fatty acids as well as enhancement of hepatic accumulation of triglycerides and low-density lipoprotein (LDL) reuptake. In addition, hypothyroidism is associated with body weight and reduced resting energy expenditure, which further favors lipid storage within the liver **[12,13]**. Finally, animal studies suggest a role of T3 in the organization of the liver microtubular network, hepatic mitochondrial turnover and autophagy, which are known to be disrupted in NAFLD **[10,14–16]**.

The growing interest toward hypothyroidism as a modifiable risk factor for NAFLD is not only justified by its high prevalence in the general population, which ranges from 0.2% to 5.3% in Europe and 0.3% and 3.7% in US **[17]**, but also by the availability of levothyroxine replacement therapy (L-T4). In addition, selective THR-β agonists have been identified as promising treatment of both NAFLD and dyslipidemia **[18]**.

Nevertheless, in clinical practice the association between NAFLD, NASH and hypothyroidism remains controversial, since results from published studies are conflicting **[5,6,8,10,12,19–24]**. Particularly, few data have been provided on the association between thyroid function and histological features associated with progressive liver disease, such as activity and fibrosis **[25]**. In fact, data on the association between hypothyroidism and activity (i.e. NAS) remain inconclusive **[26–29]**, whereas no studies have investigated the relationship between thyroid function tests and either fibrosis or steatosis.

Here, we analyzed the prevalence of hypothyroidism in a well-characterized cohort of NAFLD patients, and evaluated the association of thyroid function tests with the main histological features of NAFLD, namely activity, fibrosis and steatosis.

## Material and methods

This is a retrospective single-Center study conducted on patients consecutively submitted to liver biopsy (LB) for NAFLD between 2014 and 2016 at the Gastroenterology and Hepatology Department of the Foundation IRCCS Ca’ Granda Ospedale Maggiore Policlinico in Milan, Italy. NAFLD was defined by the presence of steatosis at ultrasound (US), when other causes of chronic liver disease (i.e. viral, autoimmune, drug-induced, vascular and inherited disorders) were excluded. Patients with significant alcohol consumption (20g/day for women and 30g/day for men) were also excluded from the analysis.

Anthropometric and clinical data were collected on the same day of LB, as well as liver stiffness measurement (LSM), through transient elastography (TE). Blood samples were concomitantly collected and served to retrospectively assess thyroid function tests (*see below*). Thyroid function tests were performed blindly on blood samples collected at the time of liver biopsy, after study approval by our Ethic Committee.

Diabetes and impaired fasting glucose (IFG) were defined according to the standard of care of the American Diabetes Association **[4]**, while dyslipidemia was defined according with the most recent guidelines of the European Society of Cardiology (ESC) and the European Atherosclerosis Society (EAS) **[30].** LDL-cholesterol was calculated by the Friedewald Equation.

Data were collected between January and March 2020. Written informed consent was obtained from each patient included in the study. The study protocol was approved by the Institutional Board of our Department (Ethical Committee Milan Area 2) and conforms to the ethical guidelines of the 1975 Declaration of Helsinki.

### Histological assessment

Liver biopsies were performed with a Menghini-like semi-automatic needle (Biomol^®^ 16G, HS^®^ Hospital Service, Latina, Italy) and were considered adequate for histological analysis if they were longer than 10 mm and/or had at least 10 portal tracts. The biopsies were all read by a single Pathologist (M.M.) who was blinded to any clinical information. NAFLD was scored according to the composite NAFLD Activity Score (NAS) **[26]**, which is comprehensive of steatosis (0–3), lobular inflammation (0–2) and ballooning (0–2). NASH was defined as NAS ≥4. Steatosis was defined mild (S1; 5–33%), moderate (S2; 34–66%) or severe (S3; >66%); S2-S3 was considered significant steatosis. Conversely, patients with NAS <4 were defined as having NAFL. Fibrosis (F) was staged according to the Kleiner classification **[27]**, as follows: F0 absence of fibrosis, F1a and F1b perisinusoidal collagen, F1c portal or periportal collagen, F2 collagen extension to zone 3, F3 bridging fibrosis and F4 cirrhosis. Significant fibrosis was defined as F ≥2 (i.e. F2-F4).

### Thyroid function assessment

Thyroid function tests [Thyroid Stimulating Hormone (TSH), free-triiodothyronine (FT3), and free-thyroxin (FT4)] as well as anti-thyroperoxidase autoantibodies (TPO-Ab) and anti-Thyroglobulin (Tg-Ab) were assessed by Cobas Roche Elecsys 600 (Roche Diagnostics GmbH, Mannheim, Germany). Reverse T3 (rT3) was measured by a competitive enzyme immunoassay (DBC-Diagnostic Biochem Canada Inc, London, Ontario, Canada). All these assessments were performed in duplicate.

TPO-Ab and Tg-Ab higher than 34 IU/ml and 115 IU/ml, respectively, were considered positive.

Hypothyroidism was defined as TSH ≥4.5 μU/l and classified as subclinical or overt hypothyroidism according with the presence of normal or reduced levels of FT4 and FT3, respectively. Euthyroid patients were further divided in two subgroups according to previous suggestion for NAFLD patients **[31]**: a) “strictly normal” TSH 0.45–2.5 and b) “borderline/high” TSH >2.5 μU/l.

### Statistical analysis

Categorical variables were reported as frequencies (percentages) and continuous variables as median (range). Categorical variables were compared using the χ2 or the Fischer’s exact tests; continuous variables were compared using the Student’s *t* test, the Mann-Whitney *U-*test or the Kruskall-Wallis test, when appropriate. All tests were 2-sided and used a significance level of 0.05. Univariate and multivariate logistic regression analyses were performed to identify factors associated with histological parameters. Quantil regression was used to correct the association between NAS and hypothyroidism for age and BMI. Data handling and analysis were performed with Stat-View package (SAS Institute Inc., Cary, NC).

## Results

### Patient population

Between 2014 and 2016, 52 patients with liver histology consistent with NAFLD fulfilled inclusion criteria for the present study. Median age was 54 (28–73) years, most (58%) were females, median BMI was 26.2 (17.9–39.8) kg/m^2^, 17 (32%) patients were overweighed and 14 (27%) obese. Most of them had altered transaminases (63%) and/or γGT (83%) values, median LSM was 7.8 (2.7–73.5) kPa. Lipid lowering drugs were taken by 7 (22%) patients with hypercholesterolemia and 4 (17%) patients with hypertriglyceridemia. Arterial hypertension was present in 21 (40%) patients. Complete patient characteristics are reported in **Table 1**.

**Table 1:** Patient characteristics (overall population).

Characteristics	n = 52
Age, years	54 (28–73)
Males	22 (42%)
BMI (Kg/m^2^)	26.2 (17.9–39.8)
Bilirubin, mg/dl	0.6 (0.2–1.6)
AST, U/l	47 (19–165)
ALT, U/l	60 (17–203)
γGT, U/l	91 (12–455)
INR	0.98 (0.88–1.28)
pCHE, U/l	8,600 (6,229–14,124)
Albumin, g/dl	4.5 (3.5–5.1)
Cholesterol, mg/dl	199 (130–373)
HDL, mg/dl	53 (27–111)
LDL, mg/dl	118 (32–273)
Triglycerides, mg/dl	156 (40–343)
Glucose, mg/dl	98 (66–204)
HbA1c, %	6 (5.0–10.5)
PLT, 10^3^/m^3^	231 (87–434)
Creatinine, mg/dl	0.80 (0.49–1.82)
Ferritin, ng/ml	198 (5–792)
CRP, mg/dl	0.2 (0–3)
TSH, μU/ml	2.34 (0.50–36.8)
fT3, pmol/l	5.4 (4.7–7.5)
rT3, ng/dl	0.1 (0.05–0.38)
fT4, pmol/l	16.3 (4.3–20.4)
TPO-Ab >34 UI/ml	3 (6%)
Tg-Ab >115 IU/ml	3 (6%)
Comorbidities	
IFG	14 (27%)
Diabetes	14 (27%)
Hypercholesterolemia	32 (62%)
Hypertriglyceridemia	23 (44%)
Arterial hypertension	21 (40%)
Hypothyroidism	14 (27%)
LSM, kPa	7.8 (2.7–73.5)
Histology	
Activity	
NAS	4 (1–7)
NAS ≥4	31 (60%)
Fibrosis	
0	13 (25%)
1	11 (21%)
2	13 (25%)
3	5 (10%)
4	10 (9%)
Steatosis	
S1	22 (42%)
S2	12 (23%)
S3	18 (35%)

Values are expressed as n (%) or median (range).

BMI: Body Mass Index; AST: Aspartate Aminotransferase; ALT: Alanine Aminotransferase; γGT: Gamma-Glutamyl Transferase; INR: International Normalized Ratio; pCHE: Pseudo-Cholinesterase; HDL: High Density Lipoprotein; LDL: Low Density Lipoprotein; HbA1c: Glycated hemoglobin; PLT: Platelets; CRP: C-Reactive Protein; TSH: Thyroid Stimulating Hormone; fT3: Triiodothyronine; rT3: Reverse T3; fT4: Free thyroxine; TPO: Thyroid Peroxidase; Tg: Thyroglobulin; Ab: Antibodies; IFG: Impaired Fasting Glucose; LSM: Liver Stiffness Measurement; NAS: NAFLD Activity Score.

### Clinical features according to histology

Median NAS was 4, ranging from 1 to 7. A definite diagnosis of NASH (NAS ≥4) was made in 31 (60%) patients. Overall, 28 (54%) patients had significant fibrosis (F2-F4) and 10 (9%) had cirrhosis; steatosis was significant (S2-S3) in 30 (58%) patients **(Tables 1 and 2)**. Compared to those with NAFL, patients with NASH were older (p = 0.008), showed lower HDL values (p = 0.05), and displayed a higher prevalence of significant fibrosis (p = 0.023) and steatosis p<0.0001) **(Table 2)**.

**Table 2:** Patient characteristics at the time of liver biopsy, according to the presence of NASH (NAS <4 *vs*. NAS ≥4), significant fibrosis (F0-F1 *vs*. F2-F4) and significant steatosis (S0-S1 *vs*. S2-S3).

	NASH			Fibrosis			Steatosis		
Characteristics	NAS < 4 (n = 21)	NAS ≥4 (n = 31)	p-value	F0-F1 (n = 24)	F2-4 (n = 28)	p-value	S1 (n = 22)	S2-3 (n = 30)	p-value
Age, years	50 (28–73)	58 (30–72)	**0.008**	52 (28–72)	58 (34–73)	**0.03**	52 (28–73)	55 (30–72)	0.34
Males	10 (48%)	12 (39%)	0.57	15 (63%)	13 (46%)	0.28	9 (41%)	13 (43%)	0.78
Body weight, Kg	72 (43–108)	72 (38–105)	0.8	68 (43–102)	81 (38–108)	**0.05**	73 (43–102)	72 (38–108)	0.62
BMI (Kg/m^2^)	24.7 (17.9–39.8)	28.1 (19.4–35.4)	0.19	23.6 (17.9–39.8)	28.3 (19.4–37.3)	**0.018**	25.0 (17.9–39.8)	28.0 (19.4–35.4)	0.32
Bilirubin, mg/dl	0.8 (0.3–1.6)	0.5 (0.2–1.4)	0.06	0.6 (0.3–1.2)	0.6 (0.2–1.6)	0.78	0.6 (0.3–1.6)	0.6 (0.2–1.4)	0.71
AST, U/l	41 (19–139)	48 (22–165)	0.29	44 (19–165)	49 (24–97)	0.29	47 (23–139)	47 (19–165)	0.83
ALT, U/l	56 (23–136)	64 (17–203)	0.15	70 (23–203)	57 (17–181)	0.91	60 (23–136)	61 (17–203)	0.53
ɣGT, U/l	85 (15–435)	167 (12–455)	0.73	84 (19–416)	132 (12–455)	0.28	83 (15–435)	165 (12–455)	**0.033**
INR	1.01 (0.88–1.20)	0.98 (0.88–1.28)	0.35	0.98 (0.88–1.15)	1.03 (0.88–1.28)	**0.041**	1.01 (0.88–1.28)	0.98 (0.88–1.20)	0.26
pCHE, U/l	8,274 (6,229–10,633)	8,837 (6,480–14,124)	0.11	8,775 (6,902–11,522)	8,369 (6,229–14,124)	0.33	8,600 (6,229–10,633)	8,585 (6,398–14,124)	0.62
Albumin, g/dl	4.6 (3.5–5.1)	4.4 (3.8–5.0)	0.21	4.6 (3.9–5.0)	4.4. (3.5–5.1)	0.16	4.6 (3.5–5.1)	4.4 (3.8–5.0)	0.4
Cholesterol, mg/dl	208 (130–302)	195 (1**33**–373)	0.38	230 (134–373)	199 (130–263)	**0.0016**	208 (130–302)	192 (133–373)	0.09
HDL, mg/dl	59 (27–111)	49 (34–88)	**0.05**	57 (35–111)	49 (27–88)	0.16	65 (27–111)	46 (34–88)	**0.0013**
LDL, mg/dl	124 (50–182)	114 (32–273)	0.4	133 (32–273)	101 (50–147)	**0.0016**	124 (50–180)	115 (32–273)	0.38
Triglycerides, mg/dl	133 (42–289)	162 (40–343)	0.36	138 (42–343)	159 (40–247)	0.9	119 (42–289)	167 (40–343)	0.14
Glucose, mg/dl	94 (67–148)	101 (66–204)	0.4	92 (67–135)	105 (66–204)	**0.0039**	94 (66–148)	101 (78–204)	0.16
HbA1c, %	5.9 (5.2–9.2)	6.1 (5.0–10.5)	0.99	5.8 (5.2–6.8)	6.1 (5.0–10.5)	0.31	6.0 (5.2–9.2)	6.0 (5.0–10.5)	0.96
PLT, 10^3^/m^3^	238 (87–355)	223 (131–434)	0.47	260 (151–403)	195 (87–434)	**0.0002**	236 (105–355)	226 (87–434)	0.5
Creatinine, mg/dl	0.80 (0.49–1.82)	0.81 (0.51–1.45)	0.99	0.79 (0.49–1.30)	0.81 (0.56–1.82)	0.42	0.76 (0.49–1.82)	0.83 (0.51–1.45)	0.26
Ferritin, ng/ml	127 (5–441)	260 (20–792)	0.07	198 (5–477)	176 (20–792)	0.67	117 (5–441)	312 (20–792)	**0.005**
CRP, mg/dl	0.10 (0.05–1.26)	0.3 (0.1–3.0)	0.41	0.1 (0–3.0)	0.2 (0–0.8)	0.38	0.1 (0–1.1)	0.3 (0–3.0)	0.36
TSH, μU/ml	2.2 (0.7–9.3)	2.4 (0.50–36.8)	0.18	2.17 (0.64–11.2)	2.38 (0.50–36.8)	0.42	2.2 (0.7–9.3)	2.3 (0.5–36.8)	0.5
fT3, pmol/l	5.5 (4.4–6.7)	5.2 (4.0–7.1)	0.63	5.6 (4.0–7.1)	5.2 (4.0–6.8)	0.33	5.3 (4.2–6.7)	5.4 (4.0–7.1)	0.68
rT3, ng/ml	0.1 (0.06–0.38)	0.1 (0.05–0.25)	0.85	0.10 (0.05–0.38)	0.10 (0.06–0.18)	0.74	0.1 (0.06–0.38)	0.1 (0.05–0.25)	0.37
fT4, pmol/l	16.3 (13.8–20.3)	16.1 (4.3–20.4)	0.76	16.2 (12.7–18.9)	16.3 (4.3–20.4)	0.69	16.3 (12.7–20.4)	15.8 (4.3–19.7)	0.44
Comorbidities							** **		
IFG/Diabetes	8 (38%)	20 (65%)	0.09	7 (29%)	20 (48%)	**0.002**	11 (50%)	9 (30%)	0.78
Hypercholesterolemia	13 (62%)	19 (61%)	0.2	20 (83%)	29 (69%)	**0.004**	16 (73%)	15 (50%)	0.15
Hypertriglyceridemia	8 (38%)	15 (48%)	0.57	9 (38%)	16 (38%)	0.41	8 (36%)	13 (43%)	0.78
Arterial hypertension	6 (29%)	15 (48%)	0.25	10 (42%)	11 (39%)	1	8 (36%)	13 (43%)	0.78
Hypothyroidism	3 (14%)	11 (35%)	0.11	4 (17%)	11 (26%)	0.21	5 (23%)	9 (30%)	0.75
LSM, kPa	5.9 (2.7–73.5)	8.0 (3.3–22.6)	0.35	5.3 (2.7–10.1)	10.8 (4.7–73.5)	**<0.0001**	6.0 (2.7–73.5)	8.0 (3.3–21.3)	0.34
Histology						** **			** **
NAS	-	-	**-**	3 81–5)	5 (1–7)	0.0006	2 (1–6)	5 (3–7)	**<0.0001**
NAS ≥4	-	-	**-**	10 (42%)	21 (75%)	**0.023**	5 (23%)	26 (87%)	**<0.0001**
F2-F4	7 (33%)	21 (68%)	**0.023**	-	-	**-**	8 (36%)	20 (67%)	**0.048**
F4	5 (24%)	5 (16%)	0.5	-	-	**-**	5 (23%)	5 (17%)	0.73
S2-S3	4 (19%)	26 (84%)	**<0.0001**	10 (42%)	21 (75%)	**0.048**	-	-	-

Values are expressed as n (%) or median (range).

NASH: Non Alcoholic Steatohepatitis; BMI: Body Mass Index; AST: Aspartate Aminotransferase; ALT: Alanine Aminotransferase; γGT: Gamma-Glutamyl Transferase; INR: International Normalized Ratio; pCHE: Pseudo-Cholinesterase; HDL: High Density Lipoprotein; LDL: Low Density Lipoprotein; HbA1c: Glycated hemoglobin; PLT: Platelets; CRP: C-Reactive Protein; TSH: Thyroid Stimulating Hormone; fT3: Triiodothyronine; rT3: Reverse T3; fT4: Free thyroxin; IFG: Impaired Fasting Glucose; LSM: Liver Stiffness Measurement; NAS: NAFLD Activity Score. F: Fibrosis; S: Steatosis.

Patients with significant fibrosis (F2-F4) were older (p = 0.03), with higher BMI (p = 0.018) and LSM (p<0.0001) values, when compared to F0-F1. Moreover, the prevalence of IFG or diabetes was higher (p = 0.002). Histologically, these patients had higher rates of NASH (p = 0.023) and steatosis (p = 0.048). Patients with significant steatosis (S2-S3) had higher ɣGT (p = 0.033) and ferritin (p = 0.005) values, and lower HDL (p = 0.0013), a higher prevalence of both NASH (p<0.0001) and significant fibrosis (p = 0.048) **(Table 2)**. Complete patients’ characteristics according to main histological features are reported in **Table 2.**

### Thyroid function tests

In the overall cohort, 38 (73%) patients were euthyroid, and 14 (27%) had hypothyroidism. Six out of hypothyroid patients (43%) were taking L-T4 replacement treatment at the time of liver biopsy, because of a previous diagnosis of primary (n = 5) or central (n = 1) hypothyroidism. The remaining eight (57%) patients were affected with newly diagnosed hypothyroidism, including one female with overt hypothyroidism **(Table 3)**.

**Table 3:** Characteristics of patients with and without hypothyroidism.

Characteristics	Hypothyroidism°(n = 14)	Euthyroidism (n = 38)	p-value*
Age, years	50 (30–73)	55 (28–72)	0.63
Males	1 (7%)	21 (55%)	**0.003**
BMI (Kg/m^2^)	26.2 (19.9–29.9)	26.2 (17.9–39.8)	0.31
AST, U/l	50 (29–165)	44 (19–139)	0.81
ALT, U/l	50 (17–203)	57 (23–181)	0.98
ɣGT, U/l	87 (19–293)	102 (12–455)	0.3
INR	0.96 (0.90–1.05)	0.99 (0.88–1.28)	0.11
pCHE, U/l	9,727 (7,560–10,743)	8,532 (6,229–11,522)	0.06
Albumin, g/dl	4.4 (3.9–4.9)	4.5 (3.8–5.1)	0.62
Cholesterol, mg/dl	169 (133–235)	207 (130–373)	**0.03**
HDL, mg/dl	46 (36–69)	54 (27–88)	0.36
LDL, mg/dl	99 (32–130)	124 (50–273)	**0.004**
Triglycerides, mg/dl	177 (95–343)	150 (40–289)	0.29
Glucose, mg/dl	101 (78–148)	95 (66–148)	0.51
PLT, 10^3^/m^3^	0.80 (0.51–1.82)	0.82 (0.49–1.30)	0.25
Creatinine, mg/dl	78 (20–351)	254 (5–792)	0.49
Ferritin, ng/ml	0.5 (0.2–1.1)	0.1 (0–3.0)	0.07
CRP, mg/dl	6.9 (4.5–36.8)	2.1 (0.6–4.1)	**0.009**
Thyroid Tests			
fT3, pmol/l	5.6 (4.0–6.8)	5.4 (4.0–7.1)	0.95
rT3, ng/dl	0.10 (0.07–0.25)	0.10 (0.05–0.38)	0.59
fT4, pmol/l	14.7 (4.3–19.7)	16.1 (8.7–18.9)	0.59
TPO-Ab >30 UI/ml	3 (21%)	1 (11%)	**0.016**
Comorbidities	** **		
IFG/Diabetes	9 (64%)	19 (50%)	0.53
Hypercholesterolemia	8 (57%)	24 (63%)	0.75
Hypertriglyceridemia	5 (36%)	18 (47%)	0.54
Arterial hypertension	6 (43%)	15 (39%)	1
LSM, kPa	8.5 (3.3–75.0)	7.1 (2.7–35.3)	0.24
Histology			** **
NAS	5 (1–6)	4 (1–6)	**0.02**
NAS ≥4	8 (89%)	20 (53%)	0.06
F2-F4	6 (67%)	18 (47%)	0.46
F4	1 (11%)	7 (18%)	1
S2-S3	7 (78%)	21 (55%)	0.28

Values are expressed as n (%) or median (range).

°8 patients on LT-4 replacement therapy.

*biochemical and histological parameters have been calculated in patients not on L-T4 replacement therapy (n = 8).

BMI: Body Mass Index; AST: Aspartate Aminotransferase; ALT: Alanine Aminotransferase; γGT: Gamma-Glutamyl Transferase; INR: International Normalized Ratio; pCHE: Pseudo-Cholinesterase; HDL: High Density Lipoprotein; LDL: Low Density Lipoprotein; PLT: Platelets; CRP: C-Reactive Protein; fT3: Triiodothyronine; rT3: Reverse T3; fT4: Free thyroxine; TPO: Thyroid Peroxidase; Ab: Antibodies; IFG: Impaired Fasting Glucose; LSM: Liver Stiffness Measurement; NAS: NAFLD Activity Score. F: Fibrosis; S: Steatosis.

Patients with hypothyroidism were mostly females (p = 0.002). As expected, they had higher CRP (C-Reactive Protein) (p = 0.009) but lower ferritin (p = 0.07) values **(Table 3)**. At histology, they displayed a higher prevalence of NASH (p = 0.06) and higher NAS score (p = 0.02). No differences in term of either fibrosis or steatosis severity were noted **(Table 3)**. The correlation between thyroid function and NAS remained significant after adjusting for age and BMI (coefficient 1.7, 95%CI 0.4–3.3 p = 0.045).

Among euthyroid patients, no significant differences were noted between subjects with TSH <2.5 μU/ml compared with those with TSH between 2.5 and 4.5 μUI/l.

### Univariate and multivariate analysis

Clinical variables significantly associated with NASH, significant fibrosis (F2-F4) and significant steatosis (S2-S3) at univariate and multivariate analysis are reported in **Table 4**. At multivariate analysis, age and significant steatosis were independently associated with NASH, IFG/diabetes and LSM were independently associated with F2-F3 and low HDL values and NASH with S2-S3 **(Table 4)**.

**Table 4:** Variables associated with NASH (NAS ≥4), significant fibrosis (F2-F4) and significant steatosis (S2-S3): Univariate and multivariate analysis by logistic regression analysis.

	Category	Univariate Analysis*		Multivariate Analysis	
Variable		OR (95% CI)	p-value	OR (95% CI)	p-value
**NASH (NAS ≥4)**					
Age, years	Continuous	1.08 (1.01–1.14)	**0.017**	1.10 (1.01–1.20)	**0.027**
Fibrosis	F0-F1 *vs*. F2-F4	4.20 (1.29–13.65)	**0.017**	-	-
Steatosis	S1 *vs*. S2-S3	22.10 (5.18–94.20)	**<0.0001**	28.92 (5.33–156.81)	**<0.0001**
**Significant Fibrosis (F2-F4)**					
Age, years	Continuous	1.07 (1.01–1.13)	**0.025**	-	-
BMI, Kg/m^2^	Continuous	1.14 (1.00–1.30)	**0.044**	-	-
Glucose, mg/dl	Continuous	1.04 (1.01–1.07)	**0.010**	-	-
IFG/Diabetes	Yes *vs*. No	7.29 (2.13–24.86)	**0.02**	39.7 (2.03–774.09)	**0.015**
PLT, mm^3^	Continuous	0.98 (0.97–0.99)	**0.004**	-	-
NASH	Yes *vs*. No	4.20 (1.29–13.66)	**0.017**	-	-
Steatosis	S1 *vs*. S2-S3	3.50 (1.10–11.10)	**0.033**	-	-
LSM, kPa	Continuous	1.71 (1.22–2.40)	**0.002**	2.65 (1.21–5.79)	**0.014**
**Significant Steatosis (S2-S3)**					
HDL, mg/dl	Continuous	0.93 (0.89–0.98)	**0.005**	0.93 (0.88–0.99)	**0.017**
NASH	Yes *vs*. No	22.10 (5.18–94.20)	**<0.0001**	31.25 (5.48–178.13)	**<0.0001**
Fibrosis	F0-F1 *vs*. F2-F4	3.50 (1.10–11.09)	**0.033**	-	-

*The following variables have been analyzed, however without being statistically significant at univariate analysis

• NASH: Gender, BMI, bilirubin, AST, ALT, γGT, INR, pCHE, albumin, cholesterol, HDL, LDL, triglycerides, creatinine, glucose, PLT, CRP, TSH, fT3, rT3, fT4, LSM, IFG, diabetes.

• F2-F4: Gender, bilirubin, AST, ALT, γGT, INR, pCHE, albumin, cholesterol, HDL, LDL, triglycerides, creatinine, CRP, TSH, fT3, rT3, fT4.

• S2-S3: Gender, BMI, bilirubin, AST, ALT, γGT, INR, pCHE, albumin, cholesterol, LDL; triglycerides, creatinine, glucose, PLT, CRP, TSH, fT3, rT3, fT4, LSM, IFG, diabetes.

NASH: Non-Alcoholic Steatohepatitis; NAS: NAFLD Activity Score; F: Fibrosis; S: Steatosis; OR: Odds Ratio; CI: Interval Confidence; BMI: Body Mass Index; AST: Aspartate Aminotransferase ALT: Alanine Aminotransferase; γGT: Gamma-Glutamyl Transferase; INR: International Normalized Ratio; pCHE: Pseudo-Cholinesterase; HDL: High Density Lipoprotein; LDL: Low Density Lipoprotein; PLT: Platelets; CRP: C-Reactive Protein; TSH: Thyroid Stimulating Hormone; fT3: Triiodothyronine; rT3: Reverse T3; fT4: Free thyroxine; IFG: Impaired Fasting Glucose; LSM: Liver Stiffness Measurement; NAS: NAFLD Activity Score. F: Fibrosis; S: Steatosis.

## Discussion

In the present study, we analyzed a well-characterized cohort of consecutive patients with histologically-proven NAFLD, and reported an univariable association between histological activity and hypothyroidism, whose prevalence was significant (27%), in line with previous observations **[6,30]** showing similar prevalence of hypothyroidism (either overt or subclinical) in NAFLD patients (15.2%-36.3%). Interestingly, in our cohort most (57%) hypothyroid patients were affected with an undiagnosed primary hypothyroidism, thus suggesting that TSH screening is recommended in patients with NAFLD.

In our study, we found that NAFLD patients with hypothyroidism compared with those who were euthyroid had significantly higher NAS values, with an increased, although not statistically significant, prevalence of NASH (89% *vs*. 53%, p = 0.06). At univariable analysis, this association remained statistically significant also after adjusting for age and BMI. Conversely, we did not find any association between hypothyroidism and the severity of either fibrosis or steatosis, in spite of a slightly increased prevalence of hypothyroidism in patients with F2-F4 and S2-S3 compared with those with lower severity scores. Our results are difficult to compare with what already published in literature. Indeed, whilst most studies compared NAFLD patients with healthy controls **[5–7]**, only few compared NAFLD patients with and without NASH (i.e. NASH *vs*. NAFL).

Two studies comparing the prevalence of hypothyroidism in NASH *vs*. NAFL patients, found contrasting results, as Mazo and colleagues found similar prevalence (15.7% *vs*. 15.2%), whilst Pagadala *et al*. reported an higher prevalence of hypothyroidism in NASH subjects (25% *vs*. 12.8%, p = 0.03) **[32,33]**. In favor of an association between NASH and thyroid function are other two studies. In fact, Carulli and colleagues reported higher TSH values in NASH *vs*. NAFL patients (p = 0.017) **[34]**, whilst Kim *et al*. analyzed the prevalence of NASH in patients without overt hypothyroidism but different TSH values, and found increased rates of NASH in 143 low-thyroid function patients (TSH ≥2.5 μU/ml; 59 euthyroid) *vs*. 282 strict-normal euthyroid patients (TSH 0.4–2.5 μU/ml) (p = 0.004) **[31]**.

In our study, we found similar prevalence of hypothyroidism (p = 0.11) as well as similar TSH (p = 0.18) values between patients with or without NASH. However, patients with untreated hypothyroidism displayed more severe NAS (p = 0.02) and higher prevalence of NASH (p = 0.06).

Surprisingly, very few data exist regarding the association between hypothyroid and fibrosis, which is the strongest predictor of disease severity also in NAFLD/NASH patients **[25]**. In our study, fibrosis was not influenced by thyroid function, independently of fibrosis severity. Conversely, Kim *et al*. in their euthyroid NAFLD cohort reported a higher prevalence of advanced fibrosis (F3-F4) in patients with low (TSH 2.5–4.5 μIU/l) *vs*. strict-normal thyroid function (TSH 0.4–2.5 μIU/l). Moreover, subclinical hypothyroidism (TSH >4.5) and low-normal thyroid function (TSH 2.5–4.5) were independent predictors of NASH and advanced fibrosis **[27]**. Additionally, some authors tried to assess the correlation between TSH and fibrosis by using non-invasive tools instead of liver biopsy, whose use is currently limited in clinical practice. In the Rotterdam study, Bano *et al*. studied nearly 9,500 NAFLD patients and reported that the risk of having liver fibrosis (LSM ≥8.0 kPa.) positively correlated with TSH levels and negatively with FT4 levels **[35]**. Conversely, Manka *et al*. found that low FT3 (but not FT4 or TSH) levels were independently associated with high LSM and NAFLD fibrosis score (NFS) **[36]**. Due to differences in study design and results, data on the association between hypothyroidism and liver fibrosis deserve further investigations.

Finally, although hypothyroidism may be associated with weight gain and overweight, no conclusive data exist on the association between thyroid dysfunction and steatosis; our study for the first time investigated the relationship between histological steatosis and thyroid function, without finding any association.

Taken together, these results validate the hypothesis that hypothyroidism might be an indirect player in the pathogenesis of NAFLD and NASH, which amplifies several features of the metabolic syndrome, such as endothelial dysfunction, inflammation and insulin resistance.

Interestingly, in our cohort, we did not find any difference between biochemical and histological data of hypothyroid patients on L-T4 or left untreated. In this context, Liu *et al* found that T4 supplementation improves NAFLD especially in patient with TSH>10 and associated dyslipidemia **[37]**, but only in half of the cases. This may suggest that since the pathogenesis of liver damage in NAFLD is complex and multifactorial, L-T4 alone only partially impacting on this condition.

On the other hand, the availability of liver-selective TH analogs with a higher affinity for the THR-β, the main THR isoform expressed in the liver, might be an interesting treatment also for hypothyroid patients with NAFLD not responsive to L-T4 alone. Among THR-agonists, Resmetirom (MGL-3196), is 28-fold more selective than T3 for THR-β *versus* THR-α **[18]**, and, consequently, it is not expected to cause any adverse events at heart or bone level. In addition, its effects are mainly intrahepatic, as suggested by the unchanged TSH levels found in treated patients. In healthy subjects it led to reductions in atherogenic lipids, including LDL, Apo-B and triglycerides **[38]**, thus supporting its current advancement in Phase 3 clinical trials.

Our conclusions should be cautiously interpreted as the limited sample size had probably an impact on our analysis and prevented us to gap the conflicting data still existing in literature on this topic. In addition, this study has been conducted in a tertiary-care center, which could have further influenced characteristics of patients included in this study. Finally, the absence of a control group (i.e. patients without NAFLD) as well as the retrospective design of the study reinforces the need for further and prospective analysis, given the heterogeneity of the results of previous studies.

Beyond these limitations, however, are the strengths of our study, which basically rely on the availability of histological examination as well as on the full clinical characterization of our cohort. In fact, in our study we tried not only to assess differences in thyroid function between patients with and without NASH (NAS < *vs*. ≥4), but also investigated the association between hypothyroidism and both histological fibrosis and steatosis. In addition to histological data, our study benefits from the extensive availability of complete clinical and biochemical information, as well as from blinded and centralized assessment/reading of both histology and thyroid function tests.

In conclusion, our data suggest that hypothyroidism might be a direct and/or indirect player in the pathogenesis of NASH/NAFLD, though prospective studies are warranted in order to better define the role of hypothyroidism in the pathogenesis of NASH and its associated histological features, as new and promising molecules acting on metabolic pathways regulated by thyroid hormones are on the way.
